# Robotic-assisted Kidney Transplantation: Our Experience and Literature Review

**DOI:** 10.1007/s40472-015-0051-z

**Published:** 2015-03-26

**Authors:** Ivo Tzvetanov, Giuseppe D’Amico, Enrico Benedetti

**Affiliations:** 1 Division of Transplantation, University of Illinois Hospital & Health Sciences System, Chicago, IL USA; 2 Department of Surgery, University of Illinois Hospital & Health Sciences System, 840 South Wood Street Suite 402, Chicago, IL 60612 USA

**Keywords:** Kidney, Transplantation, Robot, Transperitoneal approach, Extra-peritoneal approach

## Abstract

Kidney transplantation is the preferred treatment for patients with end-stage renal disease. While open surgery remains the gold standard, minimally invasive surgery has recently been introduced for the recipient undergoing kidney transplantation. Our team has employed the robotic surgical system to perform renal transplantation in obese recipients to minimize the risk of surgical site infections, with good results in terms of complications and graft and patient survival. However, others groups have performed kidney transplantation robotically in nonobese patients using different techniques. The da Vinci surgical system allows the performance of kidney transplantation under optimal operative conditions. Larger studies and long-term follow-up of recipients are required to determine the effectiveness of this approach. In this article, we describe our experience and review the development of the robotic-assisted kidney transplantation (RAKT).

## Introduction

The first successful kidney allograft transplantation in human from a living donor was performed by Joseph Murray in 1954 for which he received the Nobel Prize in 1990 [1, 2]. The success of transplantation of human kidney allograft resulted in the subsequent development of other solid organ transplantation like heart, lung, liver, etc. In the ensuing 50 years, several developments in immunology and pharmacology of transplantation occurred; however, the advancement of technical aspects of surgery was limited. With the introduction of laparoscopic living donor nephrectomy in 1995 and subsequent development of robotic-assisted surgery in the 1990s, a new progress occurred in the surgery of kidney transplantation (KT). The introduction of minimally invasive, precise surgical robotic systems, as the da Vinci surgical system (Intuitive Surgical, Inc.), has expanded possibilities for more difficult surgeries to be performed and has become especially promising in kidney transplantation. This system features permit significant operative advantage, especially when the operative field is deep and narrow, and when fine dissection and micro suturing are required [3–5]. Minimally invasive surgical technologies have shown also significant benefits, such as reduced recovery period, fewer wound complications, and smaller surgical scars.

In this article, we describe our experience and review the development of the robotic-assisted kidney transplantation (RAKT).

## University of Illinois’ Approach

The initiation of the robotic kidney transplant program at The University of Illinois at Chicago was in 2009, when we performed our first robotic-assisted transabdominal renal transplantation from a diseased donor [6]. After this first successful case, we performed series of RAKTs from living donors. Our only selection criterion was obesity (body mass index (BMI) >30). Once the recipient has been accepted for transplantation if living donor (LD) is available, the surgical approach depends on the patient’s BMI. If the patient has a BMI <30 kg/m^2^, an open surgical approach is chosen. Otherwise, if the patient is obese, with a BMI ≥30 kg/m^2^, the patient is approached by a minimally invasive robotic-assisted technique. Patients without LD are placed on the waiting list and if BMI ≥30 kg/m^2^ weight loss program is started.

Once a deceased donor becomes available, obese patients will undergo robotic-assisted surgery, and nonobese patients will undergo open surgery. Previous surgeries are not considered a contraindication to perform a robotic-assisted procedure. The only exclusion criteria are severe atherosclerosis of the iliac vessels of the recipient or the graft vessels (for a deceased donor). We did not consider age or immunologic risk status as a contraindication for RAKT.

### Surgical Technique

The patient is positioned supine with the legs on leg rests. Operating surgeon is at the console of the robot and the co-surgeon positioned at the bedside. A 7-cm upper midline incision is made, and a hand-access device is inserted. Subsequently, four additional trocars are inserted. One 12-mm port, for the 30° robotic scope, is placed at the umbilicus, and two 8-mm robotic ports for robotic arms are placed in the right upper and left lower quadrants (for right site implantation). Another 12-mm port, for the assistant, is inserted on the left on the umbilicus. At this point, the da Vinci surgical system is docked into position (from the patient’s right side) and integrated to the ports. After appropriate vascular dissection, the graft is brought into the operative field through the midline incision and appropriately oriented for implantation to the external iliac vessels.

The renal vein and artery are sutured end-to-side in a continuous fashion to the external iliac vein and artery, respectively, with 5–0 or 6–0 expanded polytetrafluoroethylene suture. The bladder is distended with saline and methylene blue in order to facilitate its dissection. The muscular layers are incised, and the bladder mucosa is prepared. The ureter is anastomosed to the bladder with running 5–0 polydioxanone suture. Upon completion of the anastomosis, the seromuscular layer is closed over the ureterocystostomy with a 4–0 polyglycolic-acid suture to create an antireflux mechanism. Placement of a double J ureteral stent depends on the surgeon’s preference. The intraoperative fluorescence vascular imaging using indocyanine green or intraoperative Doppler is used to assess the kidney perfusion.

### Kidney Biopsy

Considering the intraperitoneal location of the graft, we prefer to perform kidney biopsies under laparoscopic guidance. Ultrasound-guided biopsy potentially can cause significant bleeding. The procedure is completed under general anesthesia, and antibiotic prophylaxis is routinely given. Our preference for port positioning is one infra umbilical and one in the upper quadrant on the site of the graft. A Tru-Cut biopsy needle, usually 18 G, is introduced through the abdominal wall and directed towards the upper pole of the graft. Bleeding from biopsy sites is controlled effectively with cauterization. Patient is observed for 6 h and discharged home unless immediate initiation of treatment is needed. Using this technique, we did not observe any complications.

## Review of the Literature

The first RAKT was performed in France and reported in 2001 [7]. The recipient was undergoing a second transplant using a kidney procured from a deceased donor. The first graft had been transplanted through an open surgery. For RAKT in the left iliac fossa, the patient was placed in supine position with legs spread and flexed to allow rolling in the surgical cart. The assistant standing on the left side of the patient made an incision in the left lower quadrant and placed the self-retaining retractor after retraction of the peritoneum. During the remaining part of the procedure, the assistant surgeon’s role was to perform hemostasis, placing the vascular clamps and maintaining traction on the running sutures placed by the robot trough the incision. Besides a camera arm, two other instrument arms were used for arteriotomy, venotomy, vascular anastomosis, and ureteroneocystostomy. Cold ischemia time was 26 h and 45 min. Operative time was 178 min. Vascular anastomosis was performed in 57 min, and immediate graft function was achieved.

Despite the early success of this operative procedure, the enthusiasm for RAKT was missing, and further development did not occur until 2009, when our group reported the first full RAKT [6]. The recipient was a 29-year-old woman with a body mass index (BMI) of 41 kg/m^2^ who had been on hemodialysis for 5 years. The operative time was 223 min, and the blood loss was less than 50 cm^3^. The kidney had immediate graft function. No perioperative complications were observed, and the patient was discharged on postoperative day 5 with normal kidney function. The RAKT was performed by the technique we described above. The indication for a robotic approach was morbid obesity since higher BMI in kidney transplant recipients is associated with increased risk of surgical site infections which negatively impact graft survival [8].

In 2013, still our group [9•] published a retrospective study of a 6-month follow-up, where 28 obese patients underwent RAKT were compared to a frequency-matched cohort of 28 obese patients who underwent open kidney transplantation. BMI in the robotic group was 42.6 ± 7.8 kg/m^2^ compared to 38.1 ± 5.4 kg/m^2^ in the control group (*p* = 0.02). There were no surgical site infections (SSIs) in the robotic group, while 28.6 % (8/28) in the control group developed an SSI (*p* = 0.0004). The patient and graft survival were comparable between the two groups. Within the last 5 years, we have applied this standardized technique, over 135 robotic-assisted kidney transplants in obese recipients. The highest BMI of a transplanted patient was 58 kg/m^2^, and the mean BMI of the group was 43 kg/m^2^. We did not observe any SSIs in RAKT patients (unpublished data).

The first transabdominal RAKT in Europe was performed by Boggi et al. [10] in 2011. The recipient was a 37-year-old woman with lupus nephritis on dialysis for 32 months. She weighted 59 kg. The patient was positioned supine, with the right flank slightly elevated. A 7-cm suprapubic incision was made along the previous Pfannenstiel incision, and the hand-access device was inserted. The robot was placed on the patient’s right side, and a 0° telescope was used. Renal vessels were anastomosed by the robot to the external iliac vessels. Ureteral implantation was done through the suprapubic incision using open surgical technique. Before closure of the Pfannenstiel incision, the graft was covered by the cecum and pelvic peritoneum with an attempt to keep it in the retroperitoneal location. The warm ischemia time was 51 min, and immediate graft function was noted on release of vascular clamps. One day after transplant, the patient was ambulating and started oral intake. Pain was minimal, and no analgesia was required after 48 h.

Later, Menon et al. [11, 12•, 13] from Detroit collaborated with Ahlawat in Gurgaon, and Modi in Ahmedabad in establishing two kidney transplant programs in India. In 2014, they published a prospective study of 50 consecutive patients who underwent RAKT. The mean BMI was 24.1 kg/m^2^. The robot was docked between the split-legs of the patient in a lithotomy position. A GelPOINT port was used to seal the midline incision in the periumbilical region. The pelvic bed was cooled to 18–20 °C with the introduction of 180–240-ml ice slush via modified Toomey syringes. A temperature probe was used to continuously monitor renal cooling. The graft was inserted into the abdomen through the GelPOINT port incision. The vascular anastomosis and the ureteroneocystostomy were performed robotically. The vascular anastomosis time was 25.4 min, and the ureterovesical anastomosis time was 17.4 min. Mean intraoperative renal surface temperature was 20.3 °C. The average incision length was 6.1 cm. The kidney graft was retroperitonealized for the final position. The 6-month graft function was excellent.

During the same year, Tsai et al. [14] reported their experience with 10 patients. The kidney was placed in the retroperitoneum through the Gibson incision (7.7 ± 1.04 cm) in the iliac fossa. The robot was docked from behind the patient’s back, and the assistant surgeon stood between the two legs of the patient. The robotic arms were attached to the robotic ports and set to lift the abdominal wall about 3 cm higher. A 30° endoscope was placed over the Gibson incision. The kidney was placed into the abdomen through the Gibson incision. Vascular anastomosis was carried out by the robot. The ureteral implantation was done in an open fashion. Their mean BMI was 22.8 ± 3.5 kg/m^2^ (range 18.9–28.2 kg/m^2^). All of the patients with robotic surgery resumed oral intake and ambulation within 24 h after operations. Overall, the average post-transplant hospital stay was 13.6 ± 3.5 days.

A comparison of all four techniques is given in Table 1.

**Table 1:** Varying techniques of robotic-assisted kidney transplantation

Variable	Giulianotti and Oberholzer et al. [6, 9•]	Boggi et al. [10]	Menon et al. [12•, 13]	Tsai et al. [14]
No. of patients	28	1	50	10
Patient’s position	Supine with legs on legrest and table at 20-30° Trendelenburg position	Left lateral tilt and table with 15° Trendelenburg	Supine with lithotomy and steep Trendelenburg position	Supine with lithotomy and table with 15° Trendelenburg and tilted 15–20°
Docking of robot	Right side of patient	Right side of patient	Between two legs	Right side of patient
Incision for graft placement	Paraumbilical vertical	Suprapubic horizontal	Paraumbilical vertical	Gibson incision
Placement of camera port	Left lower quadrant, slightly left to midline	Left to midline below the level of umbilicus	Through GelPOINT at paraumbilical incision	Endoscope placed over Gibson incision
Graft placement	Transperitoneal	Initially transperitoneal shifted to extra-peritoneal for final position	Initially transperitoneal shifted to extra-peritoneal for final position	Extra-peritoneal
Patient group	Obese	Nonobese	Nonobese	Nonobese
Ureteric reimplantation	Robotic, no redocking required	Open surgery	Robotic, no redocking required	Open surgery

Rosales et al. [15] in 2010 reported on a patient underwent successful laparoscopic KT. Although this case report showed that a kidney can be transplanted laparoscopically, it does not demonstrate that this operation can be reliably duplicated by the transplant community. Laparoscopy is indeed used infrequently in operations requiring multiple vascular anastomoses because of loss of hand-eye coordination, use of long instruments, amplifying natural surgeon’s tremor, and carrying a fulcrum effect and poor ergonomics, causing surgeon’s fatigue [16].

## Discussion

Minimally invasive surgery including small-incision open kidney transplantation, laparoscopic, and RAKT are feasible. The da Vinci robotic surgical system has clearly the advantages of three-dimensional vision and control of the camera by a surgeon. Articulated instruments with a wide range of movements allow ease of suturing. Tracks of the surgeon’s movements 1300 times/s eliminates the tremor and increases the precision, essential for performing a good vascular anastomosis. However, vascular anastomosis through robotic arms can be technically demanding due to a lack of tactile feedback [17]. This issue can be overcome using an expanded polytetrafluoroethylene suture that is more resistant to grasp and less likely to brake.

Our experience demonstrate benefit of RAKT in obese recipients [9•], also in terms of graft survival (data not published) compared to open technique. It is known that obese patients with no SSI had the same kidney transplant success rate as patients with a normal BMI [8]. We strongly believe that RAKT offers a real alternative to dialysis for obese renal failure patients and may help to reduce health disparities due to end-stage renal disease in populations with a higher prevalence of obesity. Furthermore, as an evolution of our idea and considering the low success rate of medical weight loss before transplantation [18], we proposed to well-selected patients a combined approach with robotic sleeve gastrectomy and RAKT. We hypothesized that the weight loss and co-morbidity management may further improve the outcomes. The first data from our trial are under publication.

In the literature, different techniques have been proposed. Both transperitoneal and extra-peritoneal approach to kidney transplantation are feasible. Also, the incisions made for the graft placement are different. Every technique has its advantages, described by the authors.

Hand assistance, easier through an upper midline incision, could facilitate some operative steps, such as handling the graft during performance of the vascular anastomoses, could improve exposure especially in obese recipient, and could be useful in case of sudden hemorrhage. Incision in the upper abdomen also avoids a highly colonized pubic and groin areas. This type of incision, though puts the patient in a slightly higher risk to develop incisional hernia and in cases of conversion, should be extended to gain full access to iliac vessels.

Moreover, in our view, working transperitoneally avoids the traditional disadvantages of retroperitoneoscopy, such as limited working space, easy collapse during suctioning and blurred vision, while still maintaining the option of the graft placement in a retroperitoneal pocket. In our series, the graft remained in the intraperitoneal location, and we did not observe any disadvantages to this approach, although some complications such as a paratransplant hernia [19] and renal pedicle torsion [20] are possible. Furthermore, the intraperitoneal location of the graft increases the risk for complications related to standard ultrasonography-guided percutaneous kidney graft biopsy. For this reason, we have chosen to perform kidney graft biopsies by laparoscopic guidance.

Intraoperative regional hypothermia is a reasonable technique. We believe that this approach can be useful for surgeons with limited experience in robotic suturing as a protection if longer time to perform the anastomosis is needed. If the anastomosis time is kept in a 30–40-min range, intraoperative cooling of the graft may not be necessary.

For achievement of immediate function of the graft, we consider important to decrease the level of the pneumoperitoneum to below 10 mmHg. This maneuver seems to decrease the potential negative effect to the microcirculation of the graft from the higher intraperitoneal pressure.

## Conclusion

Robotic-assisted kidney transplantation is an emerging modality of minimally invasive surgery, and several surgeons are trying to perform it in different ways. Despite the enthusiasm for RAKT, the current high cost is the most prohibitive factor for its widespread use.

By achieving adequate kidney graft function and minimizing surgical complications, robotic-assisted renal transplantation gives the opportunity to the disadvantaged group of obese patients with ESRD to have more a realistic access to transplantation with good results in terms of outcome. We believe that this, in a long term, will compensate the initial coast and will prove profitable even from a financial viewpoint. However, robotic surgery in organ transplantation is a very advance application of the technique, and a level of expertise is needed in any surgeon who considers this approach. Larger studies and long-term follow-up of recipients are required to determine the effectiveness of RAKT.
